# *Borrelia burgdorferi sensu lato* prevalence in tick populations in Estonia

**DOI:** 10.1186/1756-3305-6-202

**Published:** 2013-07-09

**Authors:** Julia Geller, Lidia Nazarova, Olga Katargina, Irina Golovljova

**Affiliations:** 1 Department of Virology, National Institute for Health Development, Hiiu 42, 11619, Tallinn, Estonia; 2 Department of Gene Technology, Tallinn University of Technology, Faculty of Science, Tallinn, Estonia

**Keywords:** Ticks, *Borrelia burgdorferi* sensu lato, Ixodes, Lyme disease

## Abstract

**Background:**

Estonia is located in a unique area of co-distribution of *Ixodes ricinus* and *I*. *persulcatus*, which are the main tick vectors of *Borrelia burgdorferi* sensu lato. In the last decade, the incidence rate of Lyme borreliosis in Estonia has increased dramatically up to 115.4 per 100,000 in 2012. Here we present the first survey of the presence, the prevalence and genetic characteristics of *B*. *burgdorferi* s.l. complex spirochetes in the tick population in Estonia.

**Methods:**

During the years 2006–2009, 2833 unfed *Ixodes ricinus* and *I*. *persulcatus* were collected from 43 sites in 7 counties in mainland Estonia as well as in 10 sites on the Saaremaa Island. DNA samples from ticks were analyzed individually using nested PCR of the ribosomal 5S-23S spacer region followed by bidirectional sequencing.

**Results:**

The overall estimated prevalence of *B*. *burgdorferi* s.l was 9.7% and varied from 4.9% to 24.2% on the mainland and to 10.7% in Saaremaa Island. *Ixodes persulcatus* ticks showed significantly higher prevalence rates compared to that in *I*. *ricinus*-16.3% and 8.2%, respectively. The most prevalent genospecies was *B*. *afzelii* which was detected in 53.5% of *Borrelia*-positive ticks, followed by *B*. *garinii* and *B*. *valaisiana* with 26.2% and 5.5%, respectively. Also, *B*. *bavariensis* and *B*. *burgdorferi* s.s. DNA in single *I*. *ricinus* ticks were detected. *Borrelia afzelii*, *B*. *garinii* and *B*. *valaisiana* were detected in both tick species. Two genetic subgroups of *B*. *garinii* (NT29 and 20047) and two genetic subgroups of *B*. *afzelii* (NT28 and VS461) were found to be circulating in all studied regions as well as in both tick species, except *B*. *garinii* subgroup NT29, which was found only in *I*. *persulcatus* ticks.

**Conclusions:**

In the current study we detected the circulation of five *B*. *burgdorferi* s.l. genospecies and estimated the prevalence in ticks in different regions of Estonia. Detection and genetic characterization of *Borrelia* genospecies, especially those of public health importance, in the natural foci may help assessing high risk areas of human exposure to *B*. *burgdorferi* s.l.

## Background

Ticks are important vectors of human and animal pathogens of viral, bacterial and protozoan nature worldwide. Lyme borreliosis (LB) is the most widely spread and most frequent tick-borne bacterial disease in Europe with an estimated 65,500 human cases annually [1]. In Estonia the incidence rate of LB has increased in the last decade from 23.4 in 2002 to 115.4 per 100,000 in 2012 [2], which are the highest rates in the Baltic region [3].

The causative agents of LB are the members of *Borrelia burgdorferi* sensu lato complex spirochetes of which *B*. *afzelii*, *B*. *garinii*, *B*. *spielmanii* and *B*. *burgdorferi* sensu stricto are known to be pathogenic for humans, and *B*. *valaisiana*, and *B*. *lusitaniae* are considered potentially pathogenic [4]. The first five of these genospecies are widely spread in Europe with a predominance of *B*. *afzelii* and *B*. *garinii*[5]. It has been shown that rodents are the main reservoir for *B*. *afzelii*[6] and *B*. *bavariensis*[7], while *B*. *garinii* and *B*. *valaisiana* are associated mostly with birds [8,9] and *B*. *burgdorferi* s.s. circulates in both rodent and avian hosts [10].

Ticks of the *Ixodes* spp. are the main vectors for *Borrelia* spirochetes. *Ixodes ricinus* and *I*. *persulcatus* ticks are the main vectors of *B*. *burgdorferi* s.l. in the natural foci in Europe and Asia. Nymphs are the most important in the infection cycle, while the role of adults is insignificant, as male *Ixodes* ticks usually do not feed and female ticks prefer feeding on larger mammals that are usually not competent hosts for *Borrelia* spirochetes [11]. *Borrelia burgdorferi* s.l. is transmitted transstadially, while transovarial transmission of spirochetes from the female to her offspring is a rare event [5,12]. Larvae acquire *Borrelia* during their first bloodmeal on an infected competent reservoir hosts. After molting to nymphs they transmit the infection to new uninfected hosts while feeding. Effective transmission of *Borrelia* from infected to uninfected ticks via co-feeding has also been shown to occur without development of systemic infection in hosts [13,14].

Estonia is situated in the area of *I*. *ricinus* and *I*. *persulcatus* co-distribution providing a special setting in Eastern Europe for the study of tick-borne pathogens.

A previous report from Vormsi Island has shown the presence of *B*. *burgdorferi* s.l. genospecies in *I*. *ricinus* ticks [15]. The present study is the first survey of detection and genetic characterization of *B*. *burgdorferi* s.l. genospecies in both tick species in different regions in mainland Estonia as well as on the Saaremaa Island.

## Methods

### Tick collection

**Figure 1:**
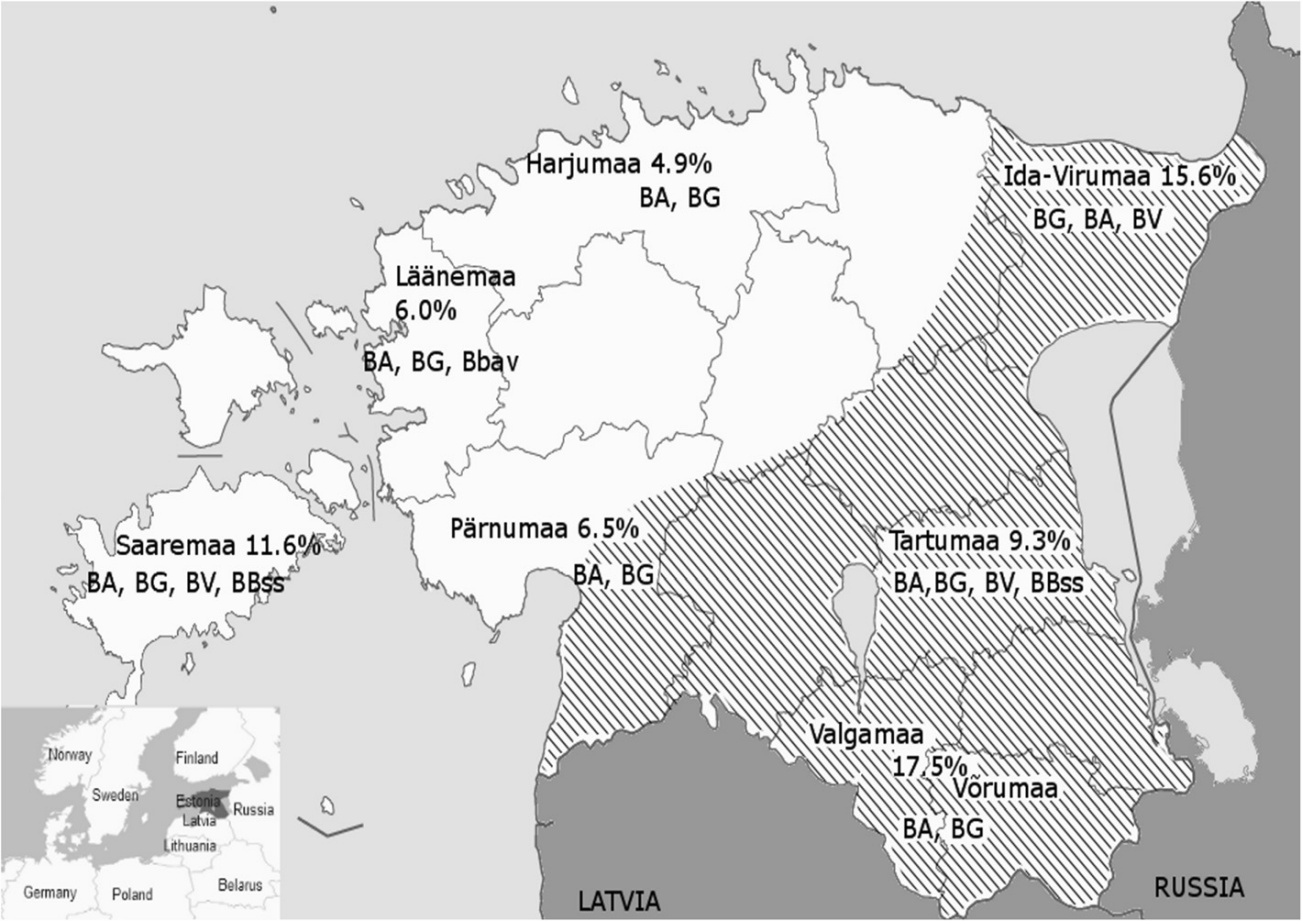
**Tick sampling sites and *B*. *burgdorferi *s.l. genospecies in Estonia.** Sites are grouped into regions according to their geographical and administrative locations. Regions are named according to their administrative location. The sympatric area for *I*. *persulcatus* and *I*. *ricinus* tick species is dashed according to Golovljova, I. unpublished data. BA- *B*. *afzelii*, BG- *B*. *garinii*, BV- *B*. *valaisiana*, Bbss- *B*. *burgdorferi* s.s., Bbav – *B*. *bavariensis*. The prevalence values of the given region are calculated according to the total tick population of the region analyzed for the presence of *B*. *burgdorferi* s.l.

Ticks were collected at 43 sites in 8 counties in mainland Estonia and 10 sites on the largest island Saaremaa during the years 2006–2009. All study sites on the mainland were grouped into 7 regions according to their geographical and administrative location, whereas 4 regions with a total of 34 sites were located in the zone of *I*. *ricinus* and *I*. *persulcatus* sympatry (Figure 1).

Questing nymphs and adult ticks of both tick species were collected by passing a 1 m^2^ flannel flag over the vegetation during the tick activity period from April until October in 2006–2009. The cloths were examined after every 5 m, all ticks were removed with forceps and maintained alive until later identification. Tick species, sex and stage were identified morphologically using a stereomicroscope. All ticks were investigated individually. Ticks were homogenized in 300 microliters of PBS with TissueLyzer (Haas, Germany). Two hundred microliters of suspension were used for DNA extraction and one hundred microliters were stored at −70°C.

### DNA extraction

The guanidinium thiocyanate-phenol-chloroform method with TriPure isolation system (Roche Diagnostics, Lewes, UK) was used for DNA extraction according to the manufacturer’s recommendations. Sterile water was included as a negative control for every DNA preparation set. DNA was kept at −20°C until further use.

### PCR amplifications

Screening of ticks was performed by PCR amplification of partial 16S rRNA genes, and the 5S-23S intergenic spacer region was used for the detection of *B*. *burgdorferi* s.l. genospecies. The presence of *Borrelia* species was detected by amplification of 16S rRNA gene as described [16] under the following conditions: 35 cycles, 94°C-10 sec, 60°C-1 min, 72°C-90 sec. Nested PCR was performed with primer pair 16S-Bor-S4F and 16S-Bor-S3R [17] and cycling conditions included 35 cycles of initial denaturation at 94°C for10 sec, annealing at 65°C-1 min, and elongation at 72°C-90 sec.

A *B*. *burgdorferi* s.l. group-specific 5S-23S intergenic spacer (IGS) region PCR was used for amplification and sequencing the 16S-positive samples as described earlier [18,19], with some modifications. Cycling conditions for the first round PCR included an initial denaturation step for 1 min at 94°C, followed by 35 cycles for 1 min at 94°C, 1 min at 58°C and 2 min at 72°C, and a final extension for 15 min at 72°C. In the nested PCR, annealing temperature was decreased to 52°C and amplification was performed for 30 cycles.

The amplified products were visualized by electrophoresis in a 1% agarose gel, stained with ethidium bromide. Negative and positive control samples were included in each step of PCR amplification. Sterile deionised water was used as negative control. Samples of *B*.*burgdorferi* s.l. genospecies DNA obtained from culture (*B*. *afzelii* strain NE632, *B*. *garinii* strain NE11, *B*. *burgdorferi* s.s. strain B31, *B*. *valaisiana* strain VS116 and *B*. *lusitaniae* strain PotiB1; kindly provided by Lise Gern) as well as from ticks, were used as positive controls. To minimize contamination risks, all steps were performed in four separate rooms using sterile techniques. The sample DNA addition step to the PCR mix was performed under laminar flow.

### DNA sequencing and phylogenetic analysis

Positive samples were sequenced with inner primers for 5S-23S IGS and 16S rRNA PCR, NC3 and NC4, and 16S-Bor-S4F and 16S-Bor-S3R, respectively. All PCR products were sent to the Estonian Biocenter (Tartu, Estonia) where sequencing was performed. Retrieved sequences were assembled, edited and analyzed with BioEdit v. 7.0.9.0 [20]. The UPGMA method was used for phylogenetic tree reconstruction using MEGA 5.0 package programs [21] with bootstrap analysis of 1000 replicates. The Maximum Likelihood Composite method was used for estimation of evolutionary distances in the units of base substitutions per site. Gamma distribution (shape parameter = 1) was used for modeling the rate variation among sites. The analysis involved 79 nucleotide sequences of 260 total positions in the final dataset.

### Statistical analysis

The 95% confidence interval (CI) of a proportion was estimated without a correction for continuity [22,23]. Statistical significance (P) of the *B*. *burgdorferi* s.l. prevalence among independent values such as tick species, stages and sites was calculated using Fisher’s exact test 2 × 2 contingency table [24] and Poisson probability test.

## Results

### Detection of *B*. *burgdorferi* DNA

A total of 2833 unfed ticks, belonging to species *I*. *ricinus* (80.9%; 2293/ 2833) or *I*. *persulcatus* (19.1%; 540/ 2833), were collected from the vegetation. *I*. *ricinus* ticks were collected in all regions of Estonia, while *I*. *persulcatus* ticks were found only in Eastern and Southern Estonia (Figure 1).

*B*. *burgdorferi* s.l. DNA was found in 275 out of 2833 ticks (9.7%) collected in all 7 regions of study. The highest overall prevalence of *B*. *burgdorferi* s.l. (15.6%- 17.7%) was detected in the South-Eastern region of Estonia (Võrumaa/Valgamaa, Ida-Virumaa), areas sympatric for *I*. *ricinus* and *I*. *persulcatus*, as well as in the allopatric area of the Saaremaa island (10.7%) where only *I*. *ricinus* is found (Table 1).

**Table 1:** ***B*. *burgdorferi *s.l. prevalence in questing ticks, collected in 7 regions in Estonia**

***I*. *ricinus***							***I*. *persulcatus***						**Prevalence,% (total No. ticks tested)**	**95% CI**
**% prevalence (No. nymphs tested)**		**95% CI**	**Prevalence,% (No. adults tested)**	**95% CI**	**Prevalence,% (total No. ticks tested)**	**95% CI**	**Prevalence,% (No. nymphs tested)**	**95% CI**	**Prevalence,% (No. adults tested)**	**95% CI**	**Prevalence,% (total No. ticks tested)**	**95% CI**		
Ida-Virumaa	9.5 (21)	2.6-28.9	4.0 (50)	1.1-13.5	5.6 (71)	2.2-13.6	10.0 (10)	1.8-40.4	23.2 (99)	16.0-32.5	22.0 (109)	15.3-30.1	15.6 (180)	11.0-21.6
Tartumaa	3.0 (67)	0.8-10.2	10.9 (230)	7.5-15.6	9.1 (297)	6.3-12.9	7.8 (102)	4.0-14.7	10.4 (192)	6.9-15.6	9.5 (294)	6.7-13.4	9.3 (591)	7.2-11.9
Võrumaa-Valgamaa	-		9.9 (121)	5.8-16.5	9.9 (121)	5.8-16.5	-		24.6 (130)	18.0-32.7	24.6 (130)	18.0-32.7	17.5 (251)	13.3-22.7
Pärnumaa	3.5 (198)	1.7-7.1	7.8 (193)	4.8-12.4	5.6 (391)	3.8-8.4	0/1 ^a^		4/6 ^a^		4/7 ^a^		6.5 (398)	4.5-9.4
***Total in sympatric area***	***3*.*8 *( *286 *)**	***2*. *2 *- *6 *.*8***	***9*.*1 *( *594 *)**	***7*. *0 *- *11 *.*7***	***7*.*4 *( *880 *) ¥**	***5*. *8 *- *9 *.*3***	***7*.*9 *( *113 *)**	***4*. *2 *- *14 *.*4***	***18*.*5 *( *79 */ *427 *)**	***15*. *1 *- *22 *.*5***	***88*/*540 *( *16 *. *3 *)¥**	***13*. *4 *- *19 *.*6***	*10*.*8 *( *1420 *)**	***9*. *3 *- *12 *.*5***
Harjumaa	3.4 (178)	1.6-7.2	6.6 (166)	3.7-11.5	4.9 (344)	3.1-7.8	-		-		-		4.9 (344)	3.1-7.8
Läänemaa	8.0 (100)	4.1-15.0	4.0 (100)	1.6-9.8	6.0 (200)	3.5-10.2	-		-		-		6.0 (200)	3.5-10.2
***Total in allopatric area*, *mainland***	***5*.*0 *( *278 *)**	***3*. *0 *- *8 *.*3***	***5*.*6 *( *266 *)**	***5*. *6 *- *14 *.*4***	***5*.*3 *( *544 *) ‡‡**	***3*. *7 *- *7 *.*5***							*5*.*3 *( *544 *)**	***3*. *7 *- *7 *.*5***
Saaremaa	9.4 (425)	7.0-12.6	11.9 (444)	9.3-15.3	10.7 (869) ‡‡	8.8-12.9	-		-		-		10.7 (869)	8.8-12.9
***Total in allopatric area***	***7*.*7 *(*703***)	***5*. *9 *- *9 *.*9***	***9*.*6 *( *710 *)**	***7*. *6 *- *12 *.*0***	***8*.*6 *( *1413 *)**	***7*. *3 *- *10 *.*2***							***8*.*6 *( *1413 *)**	***7*. *3 *- *10 *.*2***
Total	6.6 (989) ¥¥	5.2-8.3	9.4 (1304) ¥¥,‡	7.9-11.1	8.2 (2293) *	7.1-9.4	8.0 (113) ††	4.2-14.4	18.5 (427) ††,‡	15.1-22.5	16.3 (540) *	13.4-19.6	9.7 (2833)	8.7-10.9

¥,*, ‡ , **, ‡‡ Fisher’s exact and Poisson probability tests P < 0.0001.

CI-Confidence Interval of a Proportion.

a - no. of infected/ tested;% prevalence as well as 95% CI were omitted due to small number of samples.

The prevalence of *B*. *burgdorferi* s.l. in *I*. *persulcatus* ticks (16.3%) was significantly higher than that in *I*. *ricinus* (8.2%) (Table 1). The estimated infection rates within the sympatric area were twice as high in *I*. *persulcatus* ticks as in *I*. *ricinus*: 16.3% and 7.4%, respectively. However, the prevalence of *B*. *burgdorferi* s.l. in *I*. *ricinus* ticks from the sympatric areas (7.4%) did not differ much from that in allopatric areas (8.6%) (Table 1).

In mainland Estonia, the total prevalence rate of *B*. *burgdorferi* s.l. in both tick species in the sympatric area was significantly higher when compared to the prevalence rates in *I*. *ricinus* ticks in the allopatric area, 10.8% and 5.3%, respectively (P < 0.0001), while on Saaremaa Island the infection rate of *I*. *ricinus* was almost twice as high as in its mainland allopatric area (10.7% and 5.3%, respectively).

Adult ticks displayed higher infection prevalence than nymphs for both *I*. *ricinus* (9.4% for adults and 6.6% for nymphs) and *I*. *persulcatus* species (18.5% for adults and 8.0% for nymphs) (Table 1).

### Detection of *B*.*burgdorferi* s.l. genospecies

All ticks were investigated individually for the presence of *B*. *burgdorferi* s.l. by PCR amplification of partial 16S rRNA and 5S-23S IGS region genes. A total of 238 tick samples out of 275 positive for *B*. *burgdorferi* s.l. were genotyped by sequencing of PCR products and 37 samples (13.5%) contained a mix of different *B*. *burgdorferi* genospecies that could not be individually identified.

**Figure 2:**
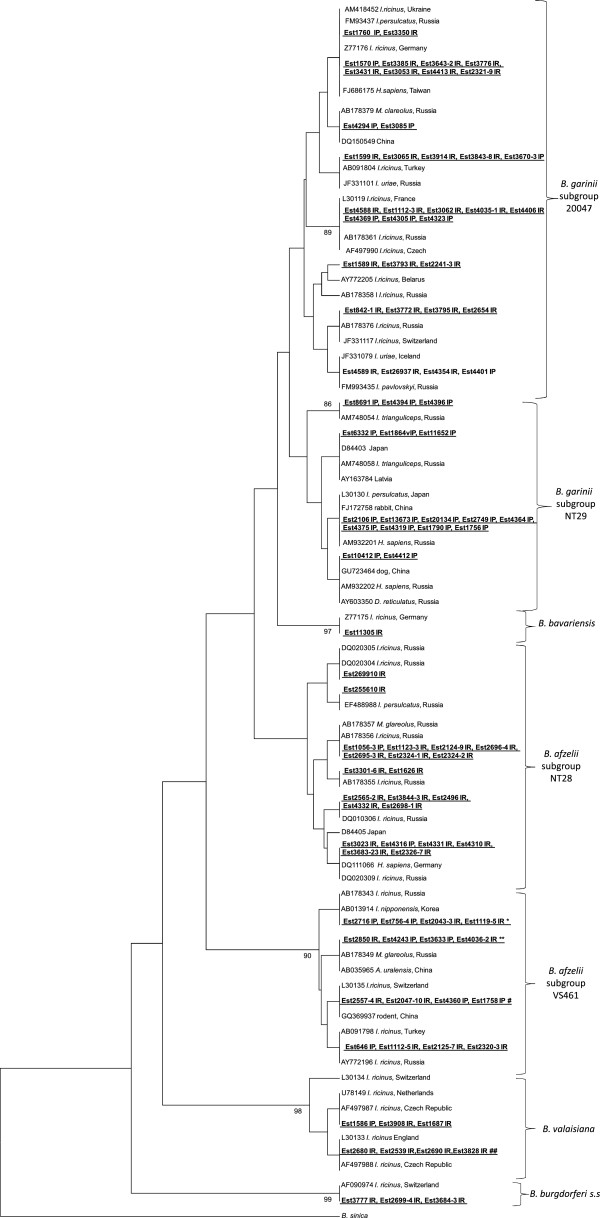
**Phylogenetic tree (UPGMA) based on the partial sequences of *B*. *burgdorferi*. s.l. 5S-23S rRNA IGS (235–253 bp).** A sequence of *B*. *sinica* retrieved from GenBank was included as the outrgroup. Only support values exceeding 70% are shown. Sequences detected in the present study are shown in bold and underlined, and followed by label of tick species from which *B*. *burgdorferi* s.l. was amplified (IR- *I*. *ricinus*, IP- *I*. *persulcatus*). *identical to Est2043-2 IR, Est2559-3 IR, Est4268 IR, Est4583 IR, Est4396 IP, Est4397 IR, Est4400 IR, Est4374 IP, Est4372 IP, Est4373 IP, Est4361 IP, Est1116-2 IR, Est2557-7 IR, Est3915 IR, Est1642 IR, Est3703-4 IP, Est3670-1 IP, Est2325-2 IR, Est1625 IR. **identical to Est4255 IP, Est633-1 IP, Est1056-2 IP, Est1635 IR, Est4317 IP, Est4301 IP, Est2254-3 IR, Est3683-20 IR, Est1644 IR, Est1165-1 IP Est771 IP, Est1828 IP, Est4036-3 IR, Est2895-7 IR, Est3697-1 IP, Est3699-3 IP, Est2848 IR, Est2700-3 IR, Est2698-5 IR, Est2696-2 IR, Est2696-1 IR, Est2694-5 IR, Est1774 IP. # identical to Est3918 IR, Est4586 IR, Est2112 IR, Est1639 IR, Est4359 IP, Est2320-7 IR, Est2319-3 IR, Est2253-1 IR, Est2244-10 IR, Est2692-2 IR, Est2304 IR, Est2031 IR, Est1758 IP. ##identical to Est3829 IR,Est3833 IR, Est3925 IR, Est 2242–9 IR, Est2696-8 IR, Est2699-9 IR, Est3840-4 IR.

*B*. *afzelii* was the most prevalent genospecies in all study regions comprising 53.5% of all *B*. *burgdorferi* s.l. infected ticks, and was detected in both *I*. *ricinus* (56.1%) and *I*. *persulcatus* (47.7%) ticks (Table 2). Analysis of nucleotide sequences showed that the investigated samples belong to two genomic subgroups of *B*. *afzelii*, VS461 and NT28 [25-27], that were detected in 62.6% and 28.6% of all *B*. *afzelii* positive ticks, respectively. The nucleotide sequences of 5S-23S IGS share a high rate of similarity (99.1%-100% for VS461 and 98.3%-100% for NT28) with other *B*. *afzelii* sequences, reported from Russia, Belarus, Sweden, Switzerland, Italy, Turkey, Korea, China, Taiwan and Japan (Figure 2). The phylogenetic analysis of 5S-23S IGS rRNA gene revealed three lineages within *B*. *afzelii* subgroup VS461 and five in subgroup NT28, albeit with a bootstrap support of less than 70%. The nucleotide sequence similarity between VS461 and NT28 subgroups ranged from 96.7% to 97.9%. In addition, 8.8% of tick samples contained a mix of *B*. *afzelii* VS461 and NT28 (Table 3).

**Table 2:** ***B*. *burgdorferi *s.l. genotypes in *I*. *ricinus *and *I*. *persulcatus *tick species**

	***I*. *ricinus***	***I*. *persulcatus***	**TOTAL**
	**No. of ticks positive/total positive (prevalence, %)**	**No. of ticks positive/total positive (prevalence, %)**	**No. of ticks positive/total positive (prevalence, %)**
*B*. *afzelii* VS461	60/187 (32.1%)	32/88 (36.4%)	92/275 (33.5%)
*B*. *afzelii* NT28	37/187 (18.8%)	5/88 (5.7%)	42/275 (15.3%)
VS461+ NT28	8/187 (4.3%)	5/88 (5.7%)	13/275 (4.7%)
Total *B*. *afzelii*	105/ 187 (56.1%)	42/ 88 (47.7%)	147/ 275 (53.5%)
*B*. *garinii* 20047	38/187 (20.3%)	9/88 (10.2%)	47/275 (17.1%)
*B*. *garinii* NT29	0/187	25/88 (28.4%)	25/275 (9.1%)
Total *B*. *garinii*	38/ 187(20.3%)	34/ 88 (38.6%)	72/ 275 (26.2%)
*B*. *valaisiana*	13/ 187 (6.9%)	2/ 88 (2.3%)	15/ 275 (5.5%)
*B*. *burgdorferi s*.*s*.	3/ 187 (1.6%)	0/ 88	3/ 275 (1.1%)
*B*. *bavariensis*	1/ 187 (0.5%)	0/ 88	1/275 (0.4%)
Mix of several genotypes	27/ 187 (14.4%)	10/ 88 (11.4%)	37/275 (13.5%)

**Table 3:** ***B*. *afzelii *and *B*. *garinii *genospecies in *I*. *ricinus *and *I*. *persulcatus *ticks**

	***I*. *ricinus***	***I*.*persulcatus***	**Total**
*B*. *afzelii* (No.147)			
VS461	60/147 (40.8%)*	32/147 (21.8%)*	92/147 (62.6%)
NT28	37/147 (25.2%)**	5/147 (3.4%)**	42/147 (28.6%)
VS461/NT28 mix	8/147 (5.4%)	5/147 (3.4%)	13/147 (8.8%)
*B*. *garinii* (No. 72)			
20047	38/72 (52.8%)†	9/72 (12.5%)†	47/72 (65.3%)
NT29	0/72	25/72 (34.7%)	25/72 (34.7%)

*, **, † Fisher’s exact test P < 0.001.

*B*. *garinii* was found in both tick species in all study regions. It was detected in 20.3% of *I*. *ricinus* and in 38.6% of *I*. *persulcatus* ticks, giving a total infection frequency of 26.2% of all *B*. *burgdorferi* s.l. infected ticks (Table 2). Estonian sequences found in the present study belong to 20047 (Eurasian) and NT29 (Asian) genomic subgroups. *B*. *garinii* group 20047 was the most prevalent (65.3%) and was detected in both *I*. *ricinu*s and *I*. *persulcatus* at 52.8% and 12.5% of *B*. *garinii* positive ticks, respectively (Table 3). *B*. *garinii* subgroup NT29 DNA was found only in *I*. *persulcatus* at 34.7% of *B*. *garinii* positive ticks. Estonian sequences of the 5S-23S IGS of the subgroup 20047 shared similarity rates of 96.8% -100%, with sequences derived from GenBank and detected in *Ixodes* ticks and also in rodents in Russia, Czech Republic, Turkey, Ukraine, Belarus, Italy, UK, Italy, France, Switzerland and China. Within subgroup NT29, the Estonian sequences were identical with those amplified from ticks, rodents and human samples from Russia, Latvia and China with similarity rates from 98.9% to 100%. Phylogenetic analysis based on 5S-23S IGS showed that Estonian samples reported in the current study are distributed within seven lineages in subgroup 20047 and five lineages in subgroup NT29 albeit with low bootstrap values (Figure 2).

*B*. *valaisiana* DNA was detected in *I*. *ricinus* ticks collected on Saaremaa Island and in both *I*. *ricinus* and *I*. *persulcatus* ticks collected in Tartumaa and Ida-Virumaa in the sympatric mainland area with an overall infection rate of 5.5% (Table 2). The phylogenetic analysis based on 5S-23S IGS showed that Estonian sequences belong to *B*. *valaisiana* group VS116 and cluster with *B*. *valaisiana* sequences reported from Netherlands, Czech Republic and UK with similarity rates from 98.8% to 100% (Figure 2).

*B*. *burgdorferi* s.s. was found in three *I*. *ricinus* ticks (1.1%) collected from Tartumaa and Saaremaa Island. Analysis of *B*. *burgdorferi* s.s. samples showed that all three Estonian sequences are identical to each other and cluster with sequences from *Ixodes* ticks and rodents from USA and Europe as well as with sequences from patients from the Czech Republic.

*B*. *bavariensis* DNA was detected in one *I*. *ricinus* tick (0.4%) collected in Läänemaa. The nucleotide sequence of 5S-23S IGS of the Estonian sample was identical to the sequence of *B*. *garinii* strain PBi from Germany, which is a prototype strain for *B*. *bavariensis*.

## Discussion

Estonia is situated in the unique area where the ranges of *I*. *ricinus* and *I*. *persulcatus* overlap in the Eastern and Southern parts. This fact may play an important role in the distribution and diversity of tick-borne pathogens. The study describes the first survey regarding the presence, the prevalence and genetic characteristics of *B*. *burgdorferi* s.l. genospecies in questing ticks collected from different sites in *I*. *ricinus* allopatric areas as well as in the areas sympatric for both tick species in Estonia.

The overall infection rate of ticks collected from vegetation (n = 2283) was 9.7% as detected by PCR. Similar results have been reported from Sweden (11%) [28], Lithuania (10.2%) [29], Belarus (9.4%) [30] and the Moscow region in Russia (13.3%) [31]. In contrast, in neighboring Latvia, which also constitutes a sympatric area of *I*. *ricinus* and *I*. *persulcatus*, the *B*. *burgdorferi* s.l. prevalence in questing ticks was as high as 25.3% [32]. In neighboring Finland the prevalence of LB spirochetes in ticks is lower (5.1%) [15], and similar to the infection rates detected in the current study on the mainland where only *I*. *ricinus* is distributed. However, the comparison of prevalence in the neighboring countries mentioned above is difficult due to the differences in sensitivity for the different methods used for *Borrelia* detection.

Both *I*. *ricinus* and *I*. *persulcatus* tick species are important vectors of *B*. *burgdorferi* s.l. in Eurasia. Moreover, it has been suggested that *I*. *persulcatus* ticks are more efficient vectors than *I*. *ricinus* for *B*. *burgdorferi* s.l. spirochetes in the natural foci [33]. This notion is in correspondence with the results of the current study and our previous investigations of *B*. *miyamotoi*[34] and tick-borne encephalitis virus (TBEV) [35], which showed significantly higher prevalences of these tick-borne pathogens (TBPs) in *I*. *persulcatus* than in *I*. *ricinus*. In addition, the reported infection rates of these TBPs from sympatric areas are higher than those from *I*. *ricinus* ranges. We suggest that in the Eastern Estonia where the *I*. *ricinus* and *I*. *persulcatus* ranges overlap, certain more favorable environmental, biotic or abiotic factors, may play a role in the enhanced of circulation of TBPs.

According to the model, based on temperature, climate and vegetation data, recently presented by Estrada-Peňa *et al*. [36], the Western coastline of Estonia has a different pattern of tick distribution compared to that of mainland Estonia. This may be one of the reasons why the prevalence of *B*. *burgdorferi* s.l. in ticks from Saaremaa Island, as well as previously reported data from Vormsi Island [15], differs from the infection rates in ticks from mainland Estonia. Similar findings have also been shown for *B*. *miyamotoi*[34]. The high prevalence rates of *B*. *burgdorferi* s.l. in ticks from Saaremaa Island are in correspondence with data on the incidence rate of Lyme borreliosis, as the annual reported numbers of LB cases in Saaremaa are the highest in Estonia during the last decade. However, there might be other factors (abundance of small and large mammals, environment, microclimate etc.) that make the island a unique area with more favorable conditions for pathogen circulation.

Although adult ticks are not considered significant in the infection cycle of *B*. *burgdorferi* s.l., infection rates in adults are higher than in nymphs, because adult ticks have fed twice on different hosts [37]. In the current study the infection rates of adult ticks vs nymphs are higher for both *I*. *ricinus* and *I*. *persulcatus*, and are in correspondence with the values reported from regions with low infection rates in Europe in the meta analysis by Rauter and Hartung [37], as well as to those from the neighboring countries Finland [15], Latvia [38], Sweden [28].

### *B*.*burgdorferi* s.l. genospecies

The presence of *B*. *afzelii* and *B*. *garinii* in Estonian ticks has been reported previously [15,39]. As in most European countries, the most prevalent *B*. *burgdorferi* s.l. genospecies in Estonian ticks were *B*. *afzelii* (53.5%) and *B*. *garinii* (26.2%) with prevalence rates in correspondence with data from neighboring regions Russia [40], Finland [41] and Latvia [38]. Several studies from Europe indicate a wide spread of B. *afzelii* and its subgroups NT28 and VS461 [19,25] in different regions of Europe and Asia as well as in different vector species [27,42]. The fact that both genetic groups of *B*. *afzelii* and even a mix of these subgroups were found in *I*. *ricinus* as well as in *I*. *persulcatus* ticks in Estonia indicates a co-circulation of these genetic subgroups and their variants in the same natural foci and a sharing of vectors and hosts with no specific limitations. The genetic variants revealed in this study within both NT28 and VS461 subgroups also support the notion of a genetic heterogeneity of the *B*. *afzelii* genospecies circulating in Europe [27,43].

*B*. *garinii* is mostly associated with avian reservoirs, especially migratory passerines that can carry infected ticks over distances and even between continents [8]. Two genetic subgroups of *B*. *garinii* have been reported: subgroup 20047 (“European”) that circulates in *I*. *ricinus* and *I*. *persulcatus* and has a wide geographical distribution over Eurasia, and subgroup NT29 (“Asian”) [19], which has never been reported from *I*. *ricinus* up to date, and thus has a more limited geographic distribution. Thus, the detection of *B*. *garinii* subgroup NT29 in *I*. *persulcatus* ticks in Eastern Estonia makes this region, as well as areas of Eastern Latvia [32], a unique region in Eastern Europe of *B*. *garinii* subgroup NT29 circulation due to distribution of its main vector, *I*. *persulcatus*. The high nucleotide sequence identity of Estonian samples to the sequences reported from European and Asian countries and the detection of at least 11 genetic variants of *B*. *garinii* within both subgroups 20047 and NT29 are in agreement with the proposed wide geographical distribution of *B*. *garinii* as well as genetic heterogeneity of this *Borrelia* species [44,45].

*B*. *valaisiana* is also associated mostly with avian reservoirs [46,47], and our previous studies reported the detection of this *Borrelia* genospecies as well as *B*. *garinii* in ticks removed from migratory passerines [48]. In the current study we report the detection of *B*. *valaisiana* in questing ticks with infection rates similar to those reported from Sweden (6%) [28] and Norway (6%) [49]. To date there have only been rare reports on the detection of *B*. *valaisiana* in *I*. *persulcatus* ticks [50,51]. However, the detection of *B*. *valaisiana* in *I*. *persulcatus* ticks presented in the current study, as well as in Latvia [52] and in the Baltic regions of Russia [53], suggests that in areas sympatric for both *I*. *ricinus* and *I*. *persulcatus*, *B*. *valaisiana* may exchange tick vectors. Our studies on TBEV [35] and *B*. *miyamotoi*[34] also revealed that under conditions of *I*. *ricinus* and *I*. *persulcatus* sympatry, these TBPs may switch to a different tick vector and utilize both tick species as vectors. The events of sharing between tick species may also lead to adaptation of TBPs to a new vector, resulting in the spread of TBPs to new areas.

In European countries, *B*. *burgdorferi* s.s. is prevalent in *I*. *ricinus* ticks at different rates. In this study we report the presence of this *Borrelia* species in the Estonian *I*. *ricinus* population for the first time, albeit at a low rate (0.1%). Although overall data presented previously in a meta analysis have shown about 16% of ticks to be infected with *B*. *burgdorferi* s.s. in Norway, Finland, Sweden and Estonia [37], data from the Baltic regions of Russia [53], Sweden [28], Latvia [38], Finland [15], and Belarus [30] revealed infection rates ranging from 0.3 to 2.1%, which correlates with the results of the current study.

In the present study we also report for the first time the presence of *B*. *bavariensis* in ticks in Estonia. This species seems to be limited to Central Europe [54] and has been reported from Switzerland [55], Austria, Germany and the Czech Republic [56]. While a high prevalence of *B*. *bavariensis*-like *Borrelia* in *I*. *persulcatus* ticks has been reported in Mongolia [57], this *Borrelia* genospecies was detected only in *I*. *ricinus* in our study.

A part of the ticks collected in the regions of this study and investigated for the presence of *B*. *burgdorferi* s.l., were also analyzed previously for *B*. *miyamotoi*, *A*. *phagocytophilum* and TBEV [34,35,58]. However, no co-infections with TBEV (628/2833) [35] or *A*. *phagocytophilum* (739/2833) [58] were found. Double infection with *B*. *miyamotoi* and *B*. *burgdorferi* s.l. was shown only for 4 ticks out of 2458, which were analyzed for both pathogens, as recently described by Geller *et al*. [34].

## Conclusions

The recent study showed the circulation of five genospecies of *B*. *burgdorferi* s.l. complex, at least four of which, *B*. *afzelii*, *B*. *garinii*, *B*. *bavariensis* and *B*. *burgdorferi* s.s., are of medical importance. This study, as well as our previous investigations [34,35], presented an exchange of TBPs between the natural tick vectors and sympatric tick species, that may indicate adaptation to a new vector species and lead to the expansion of TBPs to new geographical ranges. As the incidence rate of LB in Estonia is the highest in the Baltic regions and the annual number of LB cases remains high, the monitoring of *Borrelia* in its natural foci as well as in its natural hosts and tick vectors is of public health importance. This may also help the understanding of the ecology of TBPs and their vectors, as well as environmental, biotic and abiotic factors that may influence the abundance of ticks, prevalence of TBPs and the morbidity rates of tick-borne diseases.

## Abbreviations

LB: Lyme borreliosis; PCR: Polymerase chain reaction; rRNA: Ribosomal RNA; IGS: Internal transcribed spacer; CI: Confidence interval; TBEV: Tick-borne encephalitis virus; TBP(s): Tick-borne pathogen(s).

## Competing interests

The authors declare that they have no competing interests.

## Authors’ contributions

JG, OK and IG carried out the field collections and morphological identifications. JG, LN, OK and IG carried out diagnostic protocols. JG, LN and IG carried out genetic studies, sequence alignments and drafted the manuscript. JG and IG participated in the design of the study and performed the statistical analysis. JG, LN, OK and IG conceived the study, and participated in its design and coordination and helped to draft the manuscript. All authors read and approved the final manuscript.
